# Endoscopic repair of primary versus recurrent male unilateral inguinal hernias: Are there differences in the outcome?

**DOI:** 10.1007/s00464-015-4318-3

**Published:** 2015-07-03

**Authors:** F. Köckerling, D. Jacob, W. Wiegank, M. Hukauf, C. Schug-Pass, A. Kuthe, R. Bittner

**Affiliations:** Department of Surgery and Center for Minimally Invasive Surgery, Academic Teaching Hospital of Charité Medical School, Vivantes Hospital, Neue Bergstrasse 6, 13585 Berlin, Germany; StatConsult GmbH, Halberstädter Strasse 40 a, 39112 Magdeburg, Germany; Department of General and Visceral Surgery, German Red Cross Hospital, Lützerodestrasse 1, 30161 Hannover, Germany; Hernia Center, Winghofer Medicum, Winghofer Strasse 42, 72108 Rottenburg am Neckar, Germany

**Keywords:** Inguinal hernia, TAPP, TEP, Recurrent, Complications

## Abstract

**Introduction:**

To date, there are no prospective randomized studies that compare the outcome of endoscopic repair of primary versus recurrent inguinal hernias. It is therefore now attempted to answer that key question on the basis of registry data.

**Patients and methods:**

In total, 20,624 patients were enrolled between September 1, 2009, and April 31, 2013. Of these patients, 18,142 (88.0 %) had a primary and 2482 (12.0 %) had a recurrent endoscopic repair. Only patients with male unilateral inguinal hernia and with a 1-year follow-up were included. The dependent variables were intra- and postoperative complications, reoperations, recurrence, and chronic pain rates. The results of unadjusted analyses were verified via multivariable analyses.

**Results:**

Unadjusted analysis did not reveal any significant differences in the intraoperative complications (1.28 vs 1.33 %; *p* = 0.849); however, there were significant differences in the postoperative complications (3.20 vs 4.03 %; *p* = 0.036), the reoperation rate due to complications (0.84 vs 1.33 %; *p* = 0.023), pain at rest (4.08 vs 6.16 %; *p* < 0.001), pain on exertion (8.03 vs 11.44 %; *p* < 0.001), chronic pain requiring treatment (2.31 vs 3.83 %; *p* < 0.001), and the recurrence rates (0.94 vs 1.45 %; *p* = 0.0023). Multivariable analysis confirmed the significant impact of endoscopic repair of recurrent hernia on the outcome.

**Conclusion:**

Comparison of perioperative and 1-year outcome for endoscopic repair of primary versus recurrent male unilateral inguinal hernia showed significant differences to the disadvantage of the recurrent operation. Therefore, endoscopic repair of recurrent inguinal hernias calls for particular competence on the part of the hernia surgeon.

The proportion of recurrences in the National Swedish Hernia Registry is 11.2 % [1]. Female sex, direct inguinal hernias at the time of the primary procedure, operation for a recurrent inguinal hernia, and smoking are significant risk factors for recurrence after inguinal hernia surgery [2]. In five meta-analyses, the outcome of open repair was compared with that of endoscopic repair of recurrent inguinal hernias [3–7]. The last meta-analysis published and which included 1311 patients from six randomized controlled trials (RCTs) and five comparative studies [7] showed that the laparoscopic technique for repair of recurrent inguinal hernia was associated with less wound infection and a faster recovery to normal activity, whereas other complication rates, including the re-recurrence rate, were comparable between the open and the endoscopic approach. Laparoscopic and open procedures could be performed with equal operation time.

On the basis of the meta-analyses, the European Hernia Society recommends endoscopic inguinal hernia techniques for recurrent hernias after conventional open repair [8]. Likewise, the International Endohernia Society recommends, with a high level of evidence, TEP and TAPP for repair of recurrent hernia as the preferred alternative to tissue repair and to the Lichtenstein repair after prior anterior repair [9]. In the Consensus Development Conference of the European Association of Endoscopic Surgery, TEP and TAPP are preferred in patients with a recurrent groin hernia after open repair. Repeat endoscopic repair is only feasible when the surgeon has a high level of experience in repeat endoscopic groin hernia repair [10].

To date, there is only one prospective study, published in German language, with 338 patients comparing endoscopic repair of primary and recurrent inguinal hernias in TEP technique [11]. In the TEP repair group of recurrent inguinal hernias, a higher incidence of injury to the peritoneum and a higher occurrence of bleeding from the epigastric vessels were observed (*p* = 0.03). The postoperative complication rate was identical in the two groups, amounting to 5.1 and 5.7 %, respectively. No differences were found between the two groups on 1-year follow-up.

By analyzing data from the Herniamed Registry [12], this paper now performs such a comparison in order to get a better estimate of the perioperative and 1-year outcome of repair of primary versus recurrent hernia on the basis of a large patient sample size.

## Patients and methods

The Herniamed Registry is a multicenter, internet-based Hernia Registry [12] into which 425 participating hospitals and surgeons engaged in private practice (Herniamed Study Group) had entered data prospectively on their patients who had undergone hernia surgery. All postoperative complications occurring up to 30 days after surgery are recorded. On 1-year follow-up, postoperative complications are once again reviewed when the general practitioner and patient complete a questionnaire. This present analysis compares the prospective data collected for all male patients with a minimum age of 16 years, who had undergone elective primary or recurrent unilateral inguinal hernia repair using either transabdominal preperitoneal patch plasty (TAPP) or total extraperitoneal patch plasty (TEP).

In total, 20,624 patients were enrolled between September 1, 2009, and August 31, 2013. Of these patients, 18,142 (88.0 %) had a primary endoscopic repair and 2482 (12.0 %) had a recurrent endoscopic repair. All the patients had to have a 1-year follow-up (follow-up rate: 100 %).

The demographic and surgery-related parameters included age (years), BMI (kg/m^2^), ASA classification (I, II, III, IV) as well as EHS classification (hernia type: medial, lateral, femoral, scrotal. Defect size: grade I = < 1.5 cm, grade II = 1.5–3 cm, grade III > 3 cm) [13], and general risk factors (nicotine, COPD, diabetes, cortisone, immunosuppression, etc.). Risk factors were dichotomized, i.e., ‘yes’ if at least one risk factor is positive and ‘no’ otherwise.

The dependent variables were intra- and postoperative complication rates, number of reoperations due to complications as well as the 1-year results (recurrence rate, pain at rest, pain on exertion, and pain requiring treatment).

All analyses were performed with the software SAS 9.2 (SAS institute Inc. Cary, NY, USA) and intentionally calculated to a full significance level of 5 %, i.e., they were not corrected in respect of multiple tests, and each *p* value ≤0.05 represents a significant result. To discern differences between the groups in unadjusted analyses, Fisher’s exact test was used for categorical outcome variables, and the robust *t* test (Satterthwaite) for continuous variables.

To rule out any confounding of data caused by different patient characteristics, the results of unadjusted analyses were verified via multivariable analyses in which, in addition to primary or recurrent operation, other influence parameters were simultaneously reviewed.

To identify influence factors in multivariable analyses, the binary logistic regression model for dichotomous outcome variables was used. Estimates for odds ratio (OR) and the corresponding 95 % confidence interval based on the Wald test were given. For influence variables with more than two categories, one of the latter forms was used in each case as reference category. For age (years) the 10-year OR estimate and for BMI (kg/m^2^) the 5-point OR estimate were given. Results are presented in tabular form, sorted by descending impact.

## Results

### Unadjusted analysis

In the endoscopic recurrent operation group, the recurrent operation was performed for *n* = 1528/2482 (61.6 %) patients following the open suture technique, for *n* = 718/2482 (28.9 %) after open mesh repair, and for *n* = 233/2.482 (9.4 %) following laparoscopic mesh repair. In terms of age, those patients with recurrent operations were significantly older (*p* < 0.001). No significant difference was noted in BMI (Table 1).

**Table 1:** Age and BMI of patients with endoscopic primary versus recurrent unilateral inguinal hernia repair in men

		Operation		*p*
		Primary	Recurrent	
Age (year)	Mean ± SD	55.5 ± 15.5	59.0 ± 15.5	<0.001
BMI (kg/m^2^)	Mean ± SD	25.8 ± 3.4	26.0 ± 3.4	0.107

The unadjusted tests aimed at discerning any relationship between operation type (primary vs recurrent operation), and the categorical influence variables showed a highly significant relationship between the ASA classification, hernia size, and all EHS classifications (in each case, *p* < 0.001) (Table 2). More recurrent operations were associated with higher ASA classifications, e.g., ASA III/IV: 17.1 vs 12.3 % as well as medial (49.8 vs 36.2 %) and femoral (3.3 vs 1.8 %) EHS classifications. On the other hand, primary operations were associated with larger defect sizes, e.g., EHS grade III: 20.8 vs 17.3 % as well as with a greater number of lateral (74.0 vs 59.2 %) and scrotal (2.8 vs 1.3 %) EHS classifications.

**Table 2:** Demographic and surgery-related parameters and risk factors of patients with endoscopic primary versus recurrent unilateral inguinal hernia repair in men

	Primary op		Recurrent op		*p*
	*n*	%	*n*	%	
ASA score					
I	6231	34.35	621	25.02	<0.001
II	9680	53.36	1437	57.90	
III/IV	2231	12.30	424	17.08	
Defect size					
I	2648	14.60	453	18.25	<0.001
II	11,726	64.63	1599	64.42	
III	3768	20.77	430	17.32	
EHS medial					
Ja	6568	36.20	1235	49.76	<0.001
Nein	11,574	63.80	1247	50.24	
EHS lateral					
Ja	13,420	73.97	1469	59.19	<0.001
Nein	4722	26.03	1013	40.81	
EHS femoral					
Ja	322	1.77	83	3.34	<0.001
Nein	17,820	98.23	2399	96.66	
EHS scrotal					
Ja	502	2.77	32	1.29	<0.001
Nein	17,640	97.23	2450	98.71	
Risk factors					
Total					
Ja	4582	25.26	747	30.10	<0.001
Nein	13,560	74.74	1735	69.90	
COPD					
Ja	866	4.77	165	6.65	<0.001
Nein	17,276	95.23	2317	93.35	
Diabetes					
Ja	812	4.48	139	5.60	0.014
Nein	17,330	95.52	2343	94.40	
Aortic aneurysm					
Ja	50	0.28	17	0.68	0.002
Nein	18,092	99.72	2465	99.32	
Immunosuppression					
Ja	85	0.47	15	0.60	0.354
Nein	18,057	99.53	2467	99.40	
Corticoids					
Ja	139	0.77	21	0.85	0.627
Nein	18,003	99.23	2461	99.15	
Nikotin abusus					
Ja	2005	11.05	292	11.76	0.292
Nein	16,137	88.95	2190	88.24	
Coagulopathy					
Ja	195	1.07	36	1.45	0.103
Nein	17,947	98.93	2446	98.55	
Antiplatelet therapy					
Ja	1133	6.25	217	8.74	<0.001
Nein	17,009	93.75	2265	91.26	
Coumarin					
Ja	296	1.63	48	1.93	0.277
Nein	17,846	98.37	2434	98.07	

As regards the risk factors, global analysis, i.e., at least one risk factor, likewise revealed a highly significant difference between the primary and recurrent operation (*p* < 0.001). Of patients with recurrences, 30.1 % had at least one risk factor, while this applied to 25.3 % of patients with a primary inguinal hernia.

As regards the individual risk factors too, the corresponding rates were sometimes significantly higher for recurrent operations (Table 2).

No difference was observed in the intraoperative complication rates between endoscopic primary and recurrent operations (Table 3). Postoperative complications, complication-related reoperations as well as the recurrence rate, pain at rest, pain on exertion, and pain requiring treatment on 1-year follow-up were significantly higher after endoscopic recurrent operations than after endoscopic primary operation (Table 3).

**Table 3:** Intra- and postoperative complications, complication-related reoperations, and 1-year follow-up results of patients with endoscopic primary versus recurrent unilateral inguinal hernia repair in men

Unadjusted analysis	Primary op		Recurrent op		*p*
	*n*	%	*n*	%	
Intraoperative complications					
Yes	232	1.28	33	1.33	0.849
No	17,910	98.72	2449	98.67	
Postoperative complications					
Yes	581	3.20	100	4.03	0.036
No	17,561	96.80	2382	95.97	
Reoperation					
Yes	153	0.84	33	1.33	0.023
No	17,989	99.16	2449	98.67	
Recurrence					
Yes	170	0.94	36	1.45	0.023
No	17,972	99.06	2446	98.55	
Pain at rest					
Yes	740	4.08	153	6.16	<0.001
No	17,402	95.92	2329	93.84	
Pain on exertion					
Yes	1457	8.03	284	11.44	<0.001
No	16,685	91.97	2198	88.56	
Chronic pain requiring treatment					
Yes	419	2.31	95	3.83	<0.001
No	17,723	97.69	2387	96.17	

### Multivariable analysis

The results of multivariable analysis of the postoperative complication rates are illustrated in Table 4 (model matching *p* < 0.001). The probability of postoperative complications was essentially determined by the scrotal EHS classification (*p* < 0.001). Likewise, a highly significant impact was exerted by hernia defect sizes, age, BMI, and lateral EHS classification on onset of postoperative complications (in each case, *p* < 0.001). Scrotal EHS classification [OR 2.558 (1.845; 3.548)], larger defect size [II vs I: OR 1.603 (1.202; 2.138); III vs I: OR 2.323 (1.699; 3.177)], and higher age [10-year OR 1.133 (1.067; 1.204)] were conducive to onset of postoperative complications (Table 4).

**Table 4:** Multivariable analysis of postoperative complications

Parameter	*p* value	Category	OR	95 % CI	
EHS scrotal	<0.001	Yes versus no	2.558	1.845	3.548
Defect size	<0.001	II (1.5–3 cm) versus I (<1.5 cm)	1.603	1.202	2.138
		III (>3 cm) versus I (<1.5 cm)	2.323	1.699	3.177
Age (10-year OR)	<0.001		1.133	1.067	1.204
BMI (5-point OR)	<0.001		0.782	0.691	0.884
EHS lateral	<0.001	Yes versus no	0.645	0.499	0.834
EHS medial	0.001	Yes versus no	0.658	0.512	0.845
Operation	0.045	Primary versus recurrent	0.797	0.638	0.995
ASA score	0.067	II versus I	1.030	0.844	1.258
		III/IV versus I	1.330	1.005	1.760
Risk factors	0.798	Yes versus no	0.976	0.814	1.172
EHS femoral	0.852	Yes versus no	1.052	0.617	1.792

On the other hand, a 5-point higher BMI [5-point OR 0.782 (0.691; 0.884)] as well as a lateral EHS classification [OR 0.645 (0.499; 0.834)] reduced the risk of postoperative complications. Likewise, a medial EHS classification (OR 0.658 [0.512; 0.845; *p* = 0.001]) and primary operations [OR 0.797 (0.638; 0.995); *p* = 0.045] significantly reduced the risk of onset of a postoperative complication. With an overall prevalence of 3.3 %, there would thus be 29 postoperative complications for every 1000 primary operations compared with 36 postoperative complications for every 1000 recurrent operations.

The results of analysis of the reoperation rate are shown in Table 5 (model matching: *p* < 0.001). Here, too, scrotal EHS classification emerged as the strongest influence factor. The reoperation risk was significantly increased for scrotal EHS classification [OR 2.266 (1.204; 4.264); *p* = 0.011]. A 5-point higher BMI was shown to be preventive here with regard to the reoperation rate [5-point OR 0.745 (0.589; 0.942); *p* = 0.014]. Likewise, primary operation significantly reduced the reoperation risk [OR 0.630 (0.428; 0.927); *p* = 0.019]. With an overall reoperation rate of 0.9 %, that thus corresponds to around seven reoperations for every 1000 patients with primary operation compared with 11 reoperations for every 1000 patients with a recurrent operation.

**Table 5:** Multivariable analysis of reoperation

Parameter	*p* value	Category	OR	95 % CI	
EHS scrotal	0.011	Yes versus no	2.266	1.204	4.264
BMI (5-point OR)	0.014		0.745	0.589	0.942
Operation	0.019	Primary versus recurrent	0.630	0.428	0.927
Defect size	0.021	II (1.5–3 cm) versus I (<1.5 cm)	1.317	0.793	2.188
		III (>3 cm) versus I (<1.5 cm)	1.970	1.130	3.436
Age (10-year OR)	0.047		1.122	1.001	1.257
Risk factors	0.083	Yes versus no	1.337	0.963	1.858
ASA score	0.083	II versus I	0.821	0.563	1.197
		III/IV versus I	1.263	0.759	2.103
EHS femoral	0.462	Yes versus no	1.405	0.568	3.480
EHS lateral	0.735	Yes versus no	1.082	0.686	1.704
EHS medial	0.798	Yes versus no	0.946	0.620	1.445

Conversely, larger hernia defect sizes [III vs I: OR 1.970 (1.130; 3.436); *p* = 0.021] as well as a higher age [10-year OR 1.122 (1.001; 1.257); *p* = 0.047] significantly increased the reoperation risk.

Table 6 illustrates the results of multivariable analysis of the parameters implicated in onset of recurrences on 1-year follow-up (model matching: *p* < 0.001). Here, the BMI emerged as the strongest influence factor (*p* = 0.004). A 5-point higher BMI increased the recurrence rate [5-point OR 1.304 (1.089; 1.562)]. Likewise, medial EHS classification significantly increased the recurrence rate on follow-up [OR 1.682 (1.144; 2.471); *p* = 0.008]. The ASA status, too, had a significant effect on the recurrence rate on follow-up, something which, however, cannot be unequivocally specified in the categories (*p* = 0.039). Conversely, for a primary operation only a tendentially predictive effect could be demonstrated [OR 0.710 (0.491; 1.027); *p* = 0.069].

**Table 6:** Multivariable analysis of recurrence in 1-year follow-up

Parameter	*p* value	Category	OR	95 % CI	
BMI (5-point OR)	0.004		1.304	1.089	1.562
EHS medial	0.008	Yes versus no	1.682	1.144	2.471
ASA score	0.039	II versus I	0.955	0.675	1.352
		III/IV versus I	1.598	0.981	2.603
Operation	0.069	Primary versus recurrent	0.710	0.491	1.027
Defect size	0.171	II (1.5–3 cm) versus I (<1.5 cm)	0.702	0.483	1.022
		III (>3 cm) versus I (<1.5 cm)	0.801	0.510	1.258
EHS scrotal	0.204	Yes versus no	1.635	0.766	3.491
Risk factors	0.370	Yes versus no	0.858	0.614	1.199
Age (10-year OR)	0.649		1.025	0.921	1.140
EHS femoral	0.702	Yes versus no	1.192	0.484	2.940
EHS lateral	0.984	Yes versus no	0.996	0.670	1.480

The results of multivariable analysis of pain at rest on 1-year follow-up are summarized in Table 7 (model matching: *p* < 0.001). That was highly significantly influenced by the operation type (*p* < 0.001). A primary operation reduced the risk of pain at rest [OR 0.661 (0.550; 0.794)]. With an overall prevalence of 4.3 %, that corresponds to 35 patients with pain at rest for every 1000 primary operations compared with 51 patients with pain at rest for patients with recurrent operations.

**Table 7:** Multivariable analysis of pain at rest in 1-year follow-up

Parameter	*p* value	Category	OR	95 % CI	
Operation	<0.001	Primary versus recurrent	0.661	0.550	0.794
BMI (5-point OR)	<0.001		1.284	1.172	1.406
Defect size	<0.001	II (1.5–3 cm) versus I (<1.5 cm)	0.666	0.561	0.791
		III (>3 cm) versus I (<1.5 cm)	0.551	0.437	0.694
Age (10-year OR)	0.056		0.952	0.905	1.001
EHS femoral	0.154	Yes versus no	1.358	0.892	2.069
Risk factors	0.188	Yes versus no	1.113	0.949	1.305
EHS scrotal	0.410	Yes versus no	0.808	0.486	1.342
ASA score	0.446	II versus I	1.038	0.880	1.225
		III/IV versus I	1.177	0.909	1.523
EHS medial	0.502	Yes versus no	0.931	0.755	1.147
EHS lateral	0.676	Yes versus no	1.050	0.835	1.320

Likewise, BMI and hernia defect size had a highly significant impact (in each case, *p* < 0.001). A higher BMI increased the risk of pain at rest [5-point OR 1.284 (1.172; 1.406)]. On the other hand, a larger defect size reduced the risk of pain [II vs I: OR 0.666 (0.561; 0.791); III vs I: OR 0.551 (0.437; 0.694)].

Equally, pain on exertion on follow-up, whose results are summarized in Table 8 (model matching: *p* < 0.001), was highly significantly influenced by the operation type (*p* < 0.001).

**Table 8:** Multivariable analysis of pain on exertion in 1-year follow-up

Parameter	*p* value	Category	OR	95 % CI	
Operation	<0.001	Primary versus recurrent	0.667	0.581	0.765
Age (10-year OR)	<0.001		0.834	0.804	0.865
Defect size	<0.001	II (1.5–3 cm) versus I (<1.5 cm)	0.721	0.635	0.819
		III (>3 cm) versus I (<1.5 cm)	0.610	0.514	0.724
BMI (5-point OR)	<0.001		1.175	1.096	1.259
EHS lateral	0.149	Yes versus no	0.883	0.746	1.046
EHS scrotal	0.166	Yes versus no	0.766	0.525	1.117
ASA score	0.198	II versus I	1.062	0.943	1.195
		III/IV versus I	1.198	0.984	1.459
EHS medial	0.466	Yes versus no	0.943	0.806	1.104
Risk factors	0.605	Yes versus no	1.032	0.916	1.163
EHS femoral	0.673	Yes versus no	1.076	0.766	1.510

Conduct of a primary operation was associated with highly significantly less pain on exertion [OR 0.667 (0.581; 0.765)]. With an overall prevalence of 8.4 %, that corresponds to onset of pain on exertion in around 68 out of every 1000 patients with primary operations compared with 99 out of every 1000 patients with recurrent operations.

Likewise, age, hernia defect size, and BMI exerted a highly significant impact on pain on exertion (in each case, *p* < 0.001). In this regard, the probability of occurrence of pain on exertion declined with higher age [10-year OR 0.834 (0.804; 0.865)] as well as in the presence of larger hernias [II vs I: OR 0.721 (0.634; 0.819); III vs I: OR 0.610 (0.514; 0.724)]. Conversely, a 5-point higher BMI increased the risk of pain [5-point OR 1.175 (1.096; 1.259)].

The results of analysis of pain requiring treatment are shown in Table 9 (model matching: *p* < 0.001). There is hardly any difference between these results and those obtained for pain on exertion. Here, too, the hernia defect size, BMI, operation type, and age played a highly significant role (in each case, *p* < 0.001). A larger defect size [II vs I: OR 0.502 (0.408; 0.619); III vs I: OR 0.404 (0.299; 0.545)], primary operation [OR 0.605 (0.480; 0.763)], and older age [10-year OR 0.880 (0.825; 0.940)] reduced the risk of chronic pain requiring treatment. Conversely, the risk of pain was increased by a 5-point higher BMI [5-point OR 1.405 (1.257; 1.570)].

**Table 9:** Multivariable analysis of chronic pain requiring treatment in 1-year follow-up

Parameter	*p* value	Category	OR	95 % CI	
Defect size	<0.001	II (1.5–3 cm) versus I (<1.5 cm)	0.502	0.408	0.619
		III (>3 cm) versus I (<1.5 cm)	0.404	0.299	0.545
BMI (5-point OR)	<0.001		1.405	1.257	1.570
Operation	<0.001	Primary versus recurrent	0.605	0.480	0.763
Age (10-year OR)	<0.001		0.880	0.825	0.940
Risk factors	0.027	Yes versus no	1.258	1.026	1.542
ASA score	0.261	II versus I	1.071	0.863	1.327
		III/IV versus I	1.318	0.942	1.844
EHS femoral	0.332	Yes versus no	1.308	0.760	2.249
EHS medial	0.429	Yes versus no	0.893	0.675	1.182
EHS scrotal	0.668	Yes versus no	0.865	0.447	1.676
EHS lateral	0.960	Yes versus no	0.992	0.732	1.345

With an overall prevalence of 2.5 %, the impact of the operation type on onset of pain requiring treatment would mean that some 19 out of every 1000 patients with primary operation suffer from pain requiring treatment compared to 31 out of every 1000 patients with recurrent operation.

Analysis of the intraoperative complications (model matching: *p* > 0.001) showed that only for medial EHS classification was a significant relationship identified. Here, the risk of intraoperative complications was reduced for patients with medial EHS classification [OR 0.564 (0.372; 0.855)]. No significant impact was identified for any of the other parameters.

## Discussion

The heterogeneous nature of recurrent hernias makes RCTs in this field difficult and time-consuming, particularly when the previous repair has to be taken into consideration [1]. Accordingly, to date there are no RCTs comparing the outcome of endoscopic repair of primary versus recurrent hernias. Large hernia registries are a valuable way of obtaining information on recurrent groin hernia surgery [1].

In this present analysis of data from the Herniamed Registry [12], the outcome of endoscopic repair of 18,142 primary hernias was compared with that of 2482 recurrent inguinal hernias on the basis of the perioperative complications and the 1-year follow-up. To enhance comparability, only male unilateral inguinal hernias for which the corresponding 1-year follow-up information was available were analyzed.

Based on the Guidelines der European Hernia Society [8], the International Endohernia Society [9], and the European Association of Endoscopic Surgery [10], endoscopic repair of recurrent inguinal hernias was performed in 61.6 % of cases following previous open suture technique, in 28.9 % following previous open mesh repair, and only in 9.4 % of cases after previous endoscopic mesh repair.

The potential risk factors identified for onset of recurrences following inguinal hernia surgery were high age, higher BMI, smoking, hernia type, and certain diseases (COPD, diabetes mellitus, aortic aneurysm, immunosuppression, etc.) [2].

Certain conclusions can be drawn, with regard to onset of inguinal hernia recurrences, from the proportion of these risk factors implicated in the two comparison groups. For example, this present analysis did not identify any significant difference between the two comparison groups in terms of mean BMI, proportion of smokers, and immunosuppressed patients. However, significant differences were found between the primary and recurrent inguinal hernia groups with regard to age, proportion of patients with a history of COPD, diabetes mellitus, and aortic aneurysm as well as patients who had to take platelet aggregation inhibitors.

On comparing the perioperative outcome of endoscopic repair of primary versus recurrent male unilateral inguinal hernias, no significant difference was discerned with regard to the intraoperative complications (1.28 vs 1.33 %; *p* = 0.849), but definitely were for the postoperative complications (3.20 vs 4.03 %; *p* = 0.036) and the complication-related reoperation rates (0.84 vs 1.33 %; *p* = 0.023). Likewise, multivariable analysis confirmed that the recurrent operation, in addition to scrotal hernia, larger defect size, higher age, and higher BMI, had a negative impact on postoperative complications. That was also true for the complication-related reoperation rates. And while the differences between the two groups are significant in view of the large sample size, the absolute values clearly show that even recurrent hernias can be operated on with a very low perioperative complication rate when using an endoscopic repair technique. Accordingly, patients should be informed in an informed consent discussion that the risk associated with endoscopic inguinal hernia repair is higher for a recurrent operation compared with a primary operation.

Equally, significant differences were seen for all criteria in the results of 1-year follow-up for endoscopic primary repair of primary versus recurrent male unilateral inguinal hernias. For example, significant differences were noted in the recurrence rates (0.94 vs 1.45 %; *p* = 0.023), pain at rest (4.08 vs 6.16 %; *p* < 0.001), pain on exertion (8.03 vs 11.44 %; *p* < 0.001), and chronic pain rate requiring treatment (2.31 vs 3.83 %; *p* < 0.001). However, multivariable analysis identified the significant impact exerted by the recurrent operation on the recurrence rate only as a trend. Rather, a higher BMI value, higher ASA classification, and medial hernia classification were responsible for re-recurrence.

Multivariable analysis identified the significantly negative impact exerted by a recurrent operation on pain at rest, pain on exertion, and pain requiring treatment. Furthermore, a higher BMI value, smaller defect size, and younger age were implicated in onset of pain after endoscopic inguinal hernia repair.

The present data thus clearly demonstrate that even when an endoscopic recurrent operation is performed in accordance with the guidelines, a poorer outcome must be expected because of the previous operation.

In the vast majority of cases, this is due to the fact that even when operating in another anatomic layer for the recurrent operation only rarely is no scarring encountered from the previous operation. As such, the conditions under which a recurrent operation is conducted are generally worse than those prevailing at the time of the primary operation, i.e., not just following previous endoscopic primary hernia operations. Therefore, a recurrent operation, i.e., also following previous open suture and mesh repair, calls for a particularly experienced surgeon. Accordingly, recurrent operations should always be performed by very experienced endoscopic hernia surgeons.

In summary, this present analysis of data from the Herniamed Registry is the first such analysis to demonstrate on the basis of a large prospective patient group the differences in outcome for up to 1 year between endoscopic repair of primary and recurrent inguinal hernia. Even when proceeding in compliance with the guidelines of the international specialist societies, more unfavorable outcomes must be expected for recurrent inguinal hernia. Hence, repair of recurrent hernias calls for particular expertise on the part of the endoscopic hernia surgeons.

## Appendix: Herniamed Study Group

### Scientific Board

**Köckerling**, Ferdinand (Chairman) (Berlin); **Berger**, Dieter (Baden–Baden); **Bittner**, Reinhard (Rottenburg); **Bruns**, Christiane (Magdeburg); **Dalicho**, Stephan (Magdeburg); **Fortelny**, René (Wien); **Jacob**, Dietmar (Berlin); **Koch**, Andreas (Cottbus); **Kraft,** Barbara (Stuttgart); **Kuthe**, Andreas (Hannover); **Lippert**, Hans (Magdeburg): **Lorenz**, Ralph (Berlin); **Mayer**, Franz (Salzburg); **Moesta**, Kurt Thomas (Hannover); **Niebuhr**, Henning (Hamburg); **Peiper**, Christian (Hamm); **Pross**, Matthias (Berlin); **Reinpold**, Wolfgang (Hamburg); **Simon**, Thomas (Sinsheim); **Stechemesser**, Bernd (Köln); **Unger**, Solveig (Chemnitz).

### Participants

**Ahmetov**, Azat (Saint-Petersburg); **Alapatt,** Terence Francis (Frankfurt/Main); **Anders,** Stefan (Berlin); **Anderson**, Jürina (Würzburg); **Arndt**, Anatoli (Elmshorn); **Asperger,** Walter (Halle); **Avram**, Iulian (Saarbrücken); **Barkus**; Jörg (Velbert); **Becker**, Matthias (Freital); **Behrend**, Matthias (Deggendorf); **Beuleke,** Andrea (Burgwedel); **Berger,** Dieter (Baden–Baden); **Bittner,** Reinhard (Rottenburg); **Blaha,** Pavel (Zwiesel); **Blumberg,** Claus (Lübeck); **Böckmann,** Ulrich (Papenburg); **Böhle**, Arnd Steffen (Bremen); **Böttger,** Thomas Carsten (Fürth); **Borchert,** Erika (Grevenbroich); **Born**, Henry (Leipzig); **Brabender,** Jan (Köln); **Brauckmann,** Markus (Rüdesheim am Rhein); **Breitenbuch von**, Philipp (Radebeul); **Brüggemann**, Armin (Kassel); **Brütting**, Alfred (Erlangen); **Budzier**, Eckhard (Meldorf); **Burghardt**, Jens (Rüdersdorf); **Carus**, Thomas (Bremen); **Cejnar**, Stephan-Alexander (München); **Chirikov**, Ruslan (Dorsten); **Comman,** Andreas (Bogen); **Crescent**i, Fabio (Verden/Aller); **Dapunt**, Emanuela (Bruneck); **Decker**, Georg (Berlin); **Demmel**, Michael (Arnsberg); **Descloux,** Alexandre (Baden); **Deusch**, Klaus-Peter (Wiesbaden); **Dick**, Marcus (Neumünster); **Dieterich**, Klaus (Ditzingen); **Dietz**, Harald (Landshut); **Dittmann**, Michael (Northeim); **Dornbusch**, Jan (Herzberg/Elster); **Drummer**, Bernhard (Forchheim); **Eckermann**, Oliver (Luckenwalde); **Eckhoff,** Jörn/Hamburg); **Elger**, Karlheinz (Germersheim); **Engelhardt**, Thomas (Erfurt); **Erichsen,** Axel (Friedrichshafen); **Eucker**, Dietmar (Bruderholz); **Fackeldey**, Volker (Kitzingen); **Farke**, Stefan (Delmenhorst); **Faust**, Hendrik (Emden); **Federmann**, Georg (Seehausen); **Feichter**, Albert (Wien); **Fiedler,** Michael (Eisenberg); **Fischer**, Ines (Wiener Neustadt); **Fortelny**, René H. (Wien); **Franczak**, Andreas (Wien); **Franke**, Claus (Düsseldorf); **Frankenberg von**, Moritz (Salem); **Frehner**, Wolfgang (Ottobeuren); **Friedhoff**, Klaus (Andernach); **Friedrich,** Jürgen (Essen); **Frings**, Wolfram (Bonn); **Fritsche**, Ralf (Darmstadt); **Frommhold,** Klaus (Coesfeld); **Frunder**, Albrecht (Tübingen); **Fuhrer**, Günther (Reutlingen); **Gassler,** Harald (Villach); **Gawad**, Karim A. Frankfurt/Main); **Gerdes**, Martin (Ostercappeln); **Gilg**, Kai-Uwe (Hartmannsdorf); **Glaubitz**, Martin (Neumünster); **Glutig,** Holger (Meißen); **Gmeiner**, Dietmar (Bad Dürrnberg); **Göring**, Herbert (München); **Grebe**, Werner (Rheda-Wiedenbrück); **Grothe**, Dirk (Melle); **Gürtler**, Thomas (Zürich); **Hache**, Helmer (Löbau); **Hämmerle**, Alexander (Bad Pyrmont); **Haffner**, Eugen (Hamm); **Hain**, Hans-Jürgen (Groß-Umstadt); **Hammans**, Sebastian (Lingen); **Hampe**, Carsten (Garbsen); **Harrer**, Petra (Starnberg); **Heinzmann**, Bernd (Magdeburg); **Heise**, Joachim Wilfried (Stolberg); **Heitland**, Tim (München); **Helbling**, Christian (Rapperswil); **Hempen**, Hans-Günther (Cloppenburg); **Henneking**, Klaus-Wilhelm (Bayreuth); **Hennes**, Norbert (Duisburg); **Hermes**, Wolfgang (Weyhe); **Herrgesell**, Holger (Berlin); **Herzing**, Holger Höchstadt); **Hessler**, Christian (Bingen); **Hildebrand**, Christiaan (Langenfeld); **Höferlin**, Andreas (Mainz); **Hoffmann**, Henry (Basel); **Hoffmann**, Michael (Kassel); **Hofmann**, Eva M. (Frankfurt/Main); **Hopfe**r, Frank (Eggenfelden); **Hornung**, Frederic (Wolfratshausen); **Hügel**, Omar (Hannover); **Hüttemann**, Martin (Oberhausen); **Huhn**, Ulla (Berlin); **Hunkeler**, Rolf (Zürich); **Imdahl**, Andreas (Heidenheim); **Jacob**, Dietmar (Berlin); **Jenert**, Burghard (Lichtenstein); **Jugenheimer**, Michael (Herrenberg); **Junger**, Marc (München); **Käs**, Stephan (Weiden); **Kahraman,** Orhan (Hamburg); **Kaiser,** Christian (Westerstede); **Kaiser**, Stefan (Kleinmachnow); **Kapischke**, Matthias (Hamburg); **Karch**, Matthias (Eichstätt); **Keck**, Heinrich (Wolfenbüttel); **Keller,** Hans W. (Bonn); **Kienzle**, Ulrich (Karlsruhe); **Kipfmüller**, Brigitte (Köthen); **Kirsch**, Ulrike (Oranienburg); **Klammer**, Frank (Ahlen); **Klatt**, Richard (Hagen); **Kleemann**, Nils (Perleberg); **Klein**, Karl-Hermann (Burbach); **Kleist**, Sven (Berlin); **Klobusicky**, Pavol (Bad Kissingen); **Kneifel**, Thomas (Datteln); **Knoop**, Michael (Frankfurt/Oder); **Knotter**, Bianca (Mannheim); **Koch,** Andreas (Cottbus); **Köckerling,** Ferdinand (Berlin); **Köhler**, Gernot (Linz); **König**, Oliver (Buchholz); **Kornblum**, Hans (Tübingen); **Krämer**, Dirk (Bad Zwischenahn); **Kraft**, Barbara (Stuttgart); **Kreissl**, Peter (Ebersberg); **Krones**, Carsten Johannes (Aachen); **Kruse,** Christinan (Aschaffenburg); **Kube**, Rainer (Cottbus); **Kühlberg**, Thomas (Berlin); **Kuhn,** Roger (Gifhorn); **Kusch,** Eduard (Gütersloh); **Kuthe,** Andreas (Hannover); **Ladberg**, Ralf (Bremen); **Ladra**, Jürgen (Düren); **Lahr-Eigen**, Rolf (Potsdam); **Lainka**, Martin (Wattenscheid); **Lammers**, Bernhard J. (Neuss); **Lancee**, Steffen (Alsfeld); **Lange**, Claas (Berlin); **Laps**, Rainer (Ehringshausen); **Larusson**, Hannes Jon (Pinneberg); **Lauschke**, Holger (Duisburg); **Leher,** Markus (Schärding); **Leidl**, Stefan (Waidhofen/Ybbs); **Lenz**, Stefan (Berlin); **Lesch**, Alexander (Kamp-Lintfort); **Liedke**, Marc Olaf (Heide); **Lienert**, Mark (Duisburg); **Limberger**, Andreas (Schrobenhausen); **Limmer**, Stefan (Würzburg); **Locher**, Martin (Kiel); **Loghmanieh**, Siawasch (Viersen); **Lorenz**, Ralph (Berlin); **Mallmann**, Bernhard (Krefeld); **Manger**, Regina (Schwabmünchen); **Maurer**, Stephan (Münster); **Mayer**, Franz (Salzburg); **Mellert**, Joachim (Höxter); **Menzel**, Ingo (Weimar); **Meurer**, Kirsten (Bochum); **Meyer**, Moritz (Ahaus**)**; **Mirow**, Lutz (Kirchberg); **Mittenzwey**, Hans-Joachim (Berlin); **Mörder-Köttgen**, Anja (Freiburg); **Moesta**, Kurt Thomas (Hannover); Moldenhauer, Ingolf (Braunschweig); **Morkramer**, Rolf (Xanten); **Mosa**, Tawfik (Merseburg); **Müller**, Hannes (Schlanders); **Münzberg**, Gregor (Berlin); **Mussack,** Thomas (St. Gallen); **Neumann**, Jürgen (Haan); **Neumeuer**, Kai (Paderborn); **Niebuhr,** Henning (Hamburg); **Nölling**, Anke (Burbach); **Nostitz**, Friedrich Zoltán (Mühlhausen); **Obermaier**, Straubing); **Öz-Schmidt**, Meryem (Hanau); **Oldorf**, Peter (Usingen); **Olivieri**, Manuel (Pforzheim); **Pawelzik**, Marek (Hamburg); **Peiper**, Christian (Hamm); **Peitgen**, Klaus (Bottrop); **Pertl**, Alexander (Spittal/Drau); **Philipp**, Mark (Rostock); **Pickart**, Lutz (Bad Langensalza); **Pizzera**, Christian (Graz); **Pöllath**, Martin (Sulzbach-Rosenberg); **Possin**, Ulrich (Laatzen); **Prenzel**, Klaus (Bad Neuenahr-Ahrweiler); **Pröve**, Florian (Goslar); **Pronnet**, Thomas (Fürstenfeldbruck); **Pross**, Matthias (Berlin); **Puff**, Johannes (Dinkelsbühl); **Rabl**, Anton (Passau); **Rapp**, Martin (Neunkirchen); **Reck**, Thomas (Püttlingen); **Reinpold,** Wolfgang (Hamburg); **Reuter**, Christoph (Quakenbrück); **Richter,** Jörg (Winnenden); **Riemann**, Kerstin (Alzenau-Wasserlos); **Rodehorst**, Anette (Otterndorf); **Roehr**, Thomas (Rödental); **Roncossek**, Bremerhaven); **Roth** Hartmut (Nürnberg); **Sardoschau**, Nihad (Saarbrücken); **Sauer**, Gottfried (Rüsselsheim); **Sauer**, Jörg (Arnsberg); **Seekamp**, Axel (Freiburg); **Seelig**, Matthias (Bad Soden); **Seidel**, Hanka (Eschweiler); **Seiler**, Christoph Michael (Warendorf); **Seltmann,** Cornelia (Hachenburg); **Senkal,** Metin (Witten); **Shamiyeh**, Andreas (Linz); **Shang**, Edward (München); **Siemssen**, Björn (Berlin); **Sievers,** Dörte (Hamburg); **Silbernik**, Daniel (Bonn); **Simon**, Thomas (Sinsheim); **Sinn**, Daniel (Olpe); **Sinning**, Frank (Nürnberg); **Smaxwil**, Constatin Aurel (Stuttgart); **Schabel**, Volker (Kirchheim/Teck); **Schadd**, Peter (Euskirchen); **Schassen von**, Christian (Hamburg); **Schattenhofer**, Thomas (Vilshofen); **Scheidbach**, Hubert (Neustadt/Saale); **Schelp**, Lothar (Wuppertal); **Scherf**, Alexander (Pforzheim); **Scheyer**, Mathias (Bludenz); **Schimmelpenning,** Hendrik (Neustadt in Holstein); **Schinkel**, Svenja (Kempten); **Schmid**, Michael (Gera); **Schmid,** Thomas (Innsbruck); **Schmidt**, Rainer (Paderborn); **Schmidt**, Sven-Christian (Berlin); **Schmidt,** Ulf (Mechernich); **Schmitz**, Heiner (Jena); **Schmitz**, Ronald (Altenburg); **Schöche**, Jan (Borna); **Schoenen**, Detlef (Schwandorf); **Schrittwieser**, Rudolf/Bruck an der Mur); **Schroll**, Andreas (München); **Schultz**, Christian (Bremen-Lesum); **Schultz**, Harald (Landstuhl); **Schulze**, Frank P. Mülheim an der Ruhr); **Schumacher**, Franz-Josef (Oberhausen); **Schwab**, Robert (Koblenz); **Schwandner**, Thilo (Lich); **Schwarz**, Jochen Günter (Rottenburg); **Schymatzek**, Ulrich (Radevormwald); **Spangenberger**, Wolfgang (Bergisch-Gladbach); **Sperling**, Peter (Montabaur); **Staade**, Katja (Düsseldorf); **Staib**, Ludger (Esslingen); **Stamm**, Ingrid (Heppenheim); **Stark**, Wolfgang (Roth); **Stechemesser,** Bernd (Köln); **Steinhilper**, Uz (München); **Stengl**, Wolfgang (Nürnberg); **Stern**, Oliver (Hamburg); **Stöltzing**, Oliver (Meißen); **Stolte**, Thomas (Mannheim); **Stopinski,** Jürgen (Schwalmstadt); **Stubbe**, Hendrik (Güstrow/); **Stülzebach**, Carsten (Friedrichroda); **Tepel,** Jürgen (Osnabrück); **Terzić**, Alexander (Wildeshausen); **Teske,** Ulrich (Essen); **Thews**, Andreas (Schönebeck); **Tichomirow,** Alexej (Brühl); **Tillenburg**, Wolfgang (Marktheidenfeld); **Timmermann,** Wolfgang (Hagen); **Tomov**, Tsvetomir (Koblenz; **Train**, Stefan H. (Gronau); **Trauzettel**, Uwe (Plettenberg); **Triechelt**, Uwe (Langenhagen); **Ulcar**, Heimo (Schwarzach im Pongau); **Unger**, Solveig (Chemnitz); **Verweel**, Rainer (Hürth); **Vogel**, Ulrike (Berlin); **Voigt**, Rigo (Altenburg); **Voit**, Gerhard (Fürth); **Volkers**, Hans-Uwe (Norden); **Vossough**, Alexander (Neuss); **Wallasch**, Andreas (Menden); **Wallner**, Axel (Lüdinghausen); **Warscher,** Manfred (Lienz); **Warwas**, Markus (Bonn); **Weber**, Jörg (Köln); **Weiß**, Johannes (Schwetzingen); **Weißenbach**, Peter (Neunkirchen); **Werner**, Uwe (Lübbecke-Rahden); **Wessel,** Ina (Duisburg); **Weyhe**, Dirk (Oldenburg); **Wieber**, Isabell (Köln); **Wiesmann**, Aloys (Rheine); **Wiesner**, Ingo (Halle); **Withöft**, Detlef (Neutraubling); **Woehe**, Fritz (Sanderhausen); **Wolf**, Claudio (Neuwied); **Yildirim**, Selcuk (Berlin); **Zarras**, Konstantinos (Düsseldorf); **Zeller**, Johannes (Waldshut-Tiengen); **Zhorzel**, Sven (Agatharied); **Zuz**, Gerhard (Leipzig).
